# Skin Immunity

**DOI:** 10.1007/s00005-017-0477-3

**Published:** 2017-06-16

**Authors:** Agata Matejuk

**Affiliations:** 1 Faculty of Health Science, Wroclaw Medical University, Wrocław, Poland; 2 Faculty of Science and Technology, Karkonosze College, Jelenia Góra, Poland

**Keywords:** Immune response, Skin, Epidermis, Dermis, Hypoxia

## Abstract

Skin is the largest organ of the body with a complex network of multitude of cell types that perform plastic and dynamic cellular communication to maintain several vital processes such as inflammation, immune response including induction of tolerance and disease prevention, wound healing, and angiogenesis. Of paramount importance are immunological functions of the skin that protect from harmful exposure coming from external and internal environments. Awareness of skin immunity can provide a better comprehension of inflammation, autoimmunity, cancer, graft-versus-host disease, vaccination, and immunotherapy approaches. This paper will update on what we currently know about immune sentinels contributing to skin immunity.

## Introduction

The skin is not only a physical barrier between external and internal environments actively protecting from stress caused by injury, microbial treat, UV irradiation, and environmental toxins. For a long time, skin was envisioned only as a static shield separating from external milieu. The concept of skin immunity and skin-associated lymphoid tissue was introduced by Streilein (1983) and this concept, although with caution, has been further extended to nominate skin as a peripheral lymphoid organ (Egawa and Kabashima 2011; Ono and Kabashima 2015). Immune system within the skin is located in both major structural compartments: epidermis and dermis and consist of several important types of immunocompetent cells. Main skin-resident immune cells, Langerhans cells (LCs) together with melanocytes that produce melanin, occupy epidermis, whereas the other types of immune specialized cells such as various dendritic cell (DCs) subpopulations, macrophages, and several T cell types reside in deeper layer—dermis. The effectiveness of the skin immune system strongly depends on the close interplay and communication between immune cells and the skin environment, e.g., neighboring keratinocytes and fibroblasts. Direct functional success of the skin immunity depends also on the flexibility of dermal vessels and the lymph nodes that drain the skin. This complex network ensures the proper surveillance and communication on which the elimination of external threat relies on. Homeostasis ends when skin function and integrity are challenged and disease starts when homeostasis is irreversibly compromised.

During the last years, a new contribution in immune response for several populations of cells residing different layers of the skin has emerged. In this paper, the role of different cellular populations that may change the fate of skin immunity is discussed.

## Immune Competence in Epidermis

### Keratinocytes

Keratinocytes constitute a major structural element of outer layer of the skin and depending on the maturation level create four strata of epidermis. Besides their structural character, recent studies found unexpected role for keratinocytes in innate and adaptive immunity (Nestle et al. 2009). Modulation of the immune system and the skin immune status strongly depends on functional keratinocytes. Keratinocytes together with neutrophils and epithelial cells create a major source of antimicrobial peptides (AMPs), small cationic and amphipathic molecules, acting as a first line of defense (Harder et al. 1997; Matejuk et al. 2010). Aberrant AMPs expression leads to the development of inflammatory skin diseases and susceptibility to microbial infections. Defective function of some AMPs such as cathelicidin and β-defensins may play a role in atopic dermatitis lesions (Wollenberg et al. 2011). Decreased AMP expression leads to increased predisposition to skin infections in atopic dermatitis, whereas high expression of AMPs is observed in psoriatic lesions (de Jongh et al. 2005; Ong et al. 2002). It has been found that cathelicidin coupled with self-DNA activates plasmocytoid DCs, key players in the pathogenesis of psoriasis (Lande et al. 2007). Expression of AMPs can be upregulated by some pro-inflammatory cytokines such as interleukin IL-17 and IL-22 (Liang et al. 2006). Recently, it has been shown that the cholinergic anti-inflammatory pathway via acetylcholine downregulates AMPs (Curtis and Radek 2012). One of AMPs, a member of cathelicidin family (LL-37), produced by keratinocytes has an essential role in promoting angiogenesis and wound healing (Zanetti 2004). Injury increases levels of LL-37 and this process is dependent on active and functional form of vitamin D3 (Schauber et al. 2007). Keratinocytes express on the surface and within, endosomes Toll-like receptors (TLRs). Activation of TLRs on keratinocytes promotes Th1 responses and production of interferons (IFNs) (Miller 2008). Keratinocytes are able to generate the production of classic pro-inflammatory cytokines such as IL-1β and IL-18 via inflammasome signalling pathway (Martinon et al. 2009). Inflammasome, a pro-inflammatory machinery, was found to be activated by UV irradiation (Feldmeyer et al. 2007; Keller et al. 2008). IL-1 produced by keratinocytes can upregulate expression of intercellular adhesion molecule (ICAM)-1. Upregulation of adhesion molecules on dermal endothelial cells and MHC class II on keratinocytes and LCs facilitate leukocyte trafficking into the skin. Besides IL-1 and IL-18, keratinocytes are able to produce IL-6, IL-10, and tumor necrosis factors (TNFs). Moreover, plasticity of keratinocytes in production of chemokines and chemokine receptors empowers them to communicate and cooperate with other cell types during immune response. In disease conditions with abundant infiltration of T cells such as psoriasis, keratinocytes express several chemokine ligands such as CXCL9, CXCL10, CXCL11, and CCL20 (Albanesi et al. 2005). The latter selectively attracts precursors of LCs into epidermis (Dieu-Nosjean et al. 2000). Upregulated levels of CXCL1 and CXCL8 by keratinocytes signal neutrophils for epidermis infiltration (Albanesi et al. 2005). Studies on graft-versus-host disease revealed that keratinocytes upon stimulation with IFN-γ can express MHC class II, thus may play a role as antigen-presenting cells (APCs) for T cell infiltrates (Nickoloff and Turka 1994). Keratinocyte involvement in crosstalk with T cells is evident. Studies show that depending on the stimuli and cellular environment, keratinocytes possess ability to induce T cells activation or antigen-specific tolerization. Keratinocytes are not able to prime naïve T cells; however, they can stimulate antigen experienced CD4 and CD8 cells (Black et al. 2007). Malfunction of keratinocytes leads to pathological conditions such as autoimmunity and cancer. With upregulation of CD40 ligand on keratinocytes, the number of LCs drastically decreases and the number of dermal DC (dDC) increases with a strong effect on tolerance disruption to skin antigens (Mehling et al. 2001). Redistribution of LCs and γδ cells in skin caused by acute as well as constitutive expression of retinoic acid early transcript 1 on keratinocytes can directly lead to cancer (Jones et al. 2008; Oppenheim et al. 2005). Interestingly, Notch1 a proto-oncogene expressed by most cancers has adverse consequences in epidermis (Koch and Radtke 2007). Reduction in Notch signalling within keratinocytes leads to defective skin barrier and development of skin tumors upon exposure to UV radiation (Demehri et al. 2009).

### Langerhans’ Cells

Considering the localization of LCs which is the outer part of the skin in comparison with other types of DCs suggests their role as first line fighters. Recent studies, however, confirm the involvement of LCs in tolerogenic responses rather than those promoting inflammation (Kaplan et al. 2008; Shklovskaya et al. 2011). Suppressive effects of LCs on contact hypersensitivity depend on their IL-10 production and induction of CD4^+^ regulatory T cells (Yoshiki et al. 2009) and tolerizing CD8^+^ T cells (Gomez de Aguero et al. 2012). Nevertheless, the role of LCs in skin immune responses stays somewhat enigmatic. For a long time, lectin Langerin (CD207) that induces the formation of Birbeck granules, characteristic structures for LCs, served as a marker for human and murine LCs (Valladeau et al. 2000). Recently, Langerin has also been shown to be expressed by dDC which constitutes distinct population of cells (Ginhoux et al. 2007; Nagao et al. 2009; Poulin et al. 2007) and is characteristic for most connective and mucosal tissues and co-exists with classic DCs Langerin negative. Besides Langerin, Langerhans cells can be recognized by other markers such as CD45, MHC class II molecules, E-cadherin, and epithelial-cell adhesion molecule (Borkowski et al. 1996; Stingl et al. 1980; Tang et al. 1993). CD1a molecule is exclusively expressed on human, but not murine LCs (Romani et al. 2006) and is able to present microbial nonpeptide antigens to T cells (Hunger et al. 2004). Mannose receptor CD206 distinguishes a subset of inflammatory dendritic epidermal cells from LCs (Guttman-Yassky et al. 2007). Interesting feature of LCs in the steady state is their ability to repopulate locally independently on the circulating precursors (Stoitzner et al. 2005). Their migratory rate through the dermal lymphatic vessels to the skin-draining lymph nodes increases during inflammation (Nishibu et al. 2006). Upon stimulation, LCs elongate their dendrites that enter epidermal tight junctions to capture antigens (Kubo et al. 2009). They maturate and finally localize in the T cell area by upregulating MHC class II molecules, co-stimulatory molecules (CD40), and essential for migration CCR7 chemokine receptor (Larsen et al. 1990; Ohl et al. 2004; Pierre et al. 1997). LCs are crucial for capturing protein antigens and mediation of Th2 local environment. Permanent LCs depletion results in decreased IgE serum levels (Nakajima et al. 2012). LCs have been found to be superior to dDCs in effective CD70-mediated CD8^+^ T cells responses to virus, with little or no effect on bacteria (van der Aar et al. 2011). However, it seems that both LCs and dDCs can resemble each other upon inflammatory reactions (Noordegraaf et al. 2010; Zahner et al. 2011) and the outcome of immune response most likely depends on the number and not the type of DCs (Romani et al. 2010). Except for hypersensitivity skin inflammation models such as cutaneous leishmaniasis, LCs are negative immune regulators (Kautz-Neu et al. 2011). The most recent studies show that in case of *Candida albicans* infection, the morphology of the pathogen dictates the proper responses by LCs or dDC subsets (Igyarto et al. 2011; Kashem et al. 2015).

## Immune Competence in Dermis

### Dermal DCs

In contrast to LCs that occupy epidermis, dDCs reside in dermis below the epidermal–dermal junction, and are distinguished by the expression of epithelial-cell adhesion molecule, IL-10, and ability to stimulate B cells into plasma cells secreting IgM (Dubois et al. 1999). Expression of low-density lipoprotein-related protein 1 (or CD91) is also characteristic for dDCs (Boyman et al. 2005). The plasticity of dDCs is remarkable and depending on the function, sublocalization, and environment, they create phenotypically diverse group of cells (Henri et al. 2010; Malissen et al. 2014; Tamoutounour et al. 2013). The main two types of dDCs are: Langerin^+^CD103^+^ DCs, which are similar to mouse CD8^+^ cross-presenting DCs in lymphoid organs (Bedoui et al. 2009) and Langerin negative dDCs. Within the latter, at least three DCs populations were identified in the murine dermis: monocytes-derived DCs with CCR2^+^CD64^low/+^ phenotype and two populations of dermal conventional DCs that originate from blood-borne precursors: a subpopulation with CD11b expression and the double negative XCR1^−^CD11b^−^ subpopulation (Auffray et al. 2009; Lopez-Bravo and Ardavin 2008). Dermal DCs can persist in immature state with expression of TLR2, TLR4, CD206, and CD209 (Angel et al. 2007) or mature state with expression of CD83 co-stimulatory molecule and low levels of TLRs. Main role for dDCs is to provide immunosurveillance against pathogens by participation in inflammatory responses via arranging efficient cytokine and chemokine network (Guttman-Yassky et al. 2007). DCs that produce both TNF-α and iNOS might play a major role in psoriasis induction (Lowes et al. 2005; Serbina et al. 2003). In addition, the positive correlation between IL-23/IL-17/IL-22 axis and psoriasis development has been demonstrated (Krueger et al. 2007; Leonardi et al. 2008; Zaba et al. 2007a). However, there is a controversy which subset of DCs is a key player in psoriasis (Glitzner et al. 2014; Wohn et al. 2013; Yoshiki et al. 2014). Tortola et al. (2012) identified IL-36, a novel member of IL-1 family that allows DCs-keratinocytes crosstalk during IL-23/IL-17/IL-22-dependent immune responses. It has also been found that in psoriasis, an important role plays LL37, an AMP that breaks tolerance to self-DNA (Lande et al. 2007). This triggers (infrequent in healthy skin) plasmocytoid DCs, strong producers of type I IFNs (Nestle et al. 2005). In consequence, myeloid DCs are activated and adaptive immune responses are induced (Boyman et al. 2007). It has been found that DCs and tissue-resident macrophages have common precursors. Inflammatory monocytes with CD115^+^LY6C^+^LY6G^+^CCR2^+^ phenotype differentiate into inflammatory DCs and monocytes with LY6C^−^LY6G^−^CX3CR1^+^ phenotype transform into activated macrophages in mice; in humans, inflammatory monocytes belong to CD14^+^CD16^−^ circulating monocytes (Auffray et al. 2009). A subpopulation of dDCs, macrophage-like cells expressing factor XIIIa and CD163 typical for macrophages, might play a key role in wound healing (Zaba et al. 2007b). The complexity of the network created by dendritic cells, monocytes, and macrophages in the skin assures effective immunosurveillance and highly diverse immune response.

### Mast Cells

Mast cells are mainly located in the upper dermal part of the skin, where they can easily encounter, respond, and protect from infections, venoms, and stress caused by wound healing. Mast cells contain histamine and traditionally are known as typical allergy cells. Nevertheless, recent studies prove their remarkable internal and external plasticity and critical role in vital processes such as wound healing, skin inflammation, angiogenesis, immune tolerance, and cancer (Galli and Tsai 2010; Moon et al. 2010; Tsai et al. 2011). In the human skin, there is a prevalence of mast cells TC type (tryptase positive, chymase positive) which is the richest in proteinase content. Besides tryptase, they contain chymase, carboxypeptidase, and a cathepsin G-like proteinase (Douaiher et al. 2014; Weidner and Austen 1993). Tryptase works on fibronectin and by degrading extracellular matrix proteins (Kaminska et al. 1999) allows immune cells such as neutrophils, mononuclear cells, and lymphocytes to invade epidermis. Its function in the activation and recruitment of the immune-competent cells (Li and He 2006; Malamud et al. 2003; Wang and He 2006) is further confirmed by its activatory effects on keratinocytes and metalloproteinases (Buddenkotte et al. 2005; Iddamalgoda et al. 2008; Sharlow et al. 2000). The enzyme has also strong proangiogenic activity (Blair et al. 1997). Similar pro-inflammatory action is characteristic for another potent mast cell enzyme, chymase. Chymase has been shown to attract and activate several immune cells (He and Walls 1998; Terakawa et al. 2006) and increase inflammation by its effect on IL-1β and IL-18 (Mizutani et al. 1991; Omoto et al. 2006). In addition to mast cells plasticity, both enzymes were found to down-regulate immune response by ability to disintegrate several pro-inflammatory factors such as cytokines and chemokines (Pang et al. 2006; Zhao et al. 2005). Besides indirect modulation of immune response by mast cells via secreted enzymes, they also affect immune-competent cells by direct cell–cell contact or cytokines. Their strong influence on different subpopulations of T cells including regulatory T cells (Treg) was observed via mast cells expression of OX-40L and TNF-α production (Nakae et al. 2005; Piconese et al. 2009). OX-40L positive mast cells and their production of TNF-α in combination with T cells derived IL-6 create classic milieu for tissue inflammation. In chronic skin inflammation such as psoriatic lesion and atopic dermatitis, mast cells secrete other cytokines such as IL-4 and/or IFN-γ that shape the immune response (Ackermann et al. 1999; Horsmanheimo et al. 1994). In cancer, mast cells can express CD30L (Molin et al. 2001) leading to uncontrolled immune response. Besides known FcεRI receptor involved in allergic response, skin mast cells also express FcγRI and FcγRIIa receptors involved in IgG responses (Malbec and Daeron 2007; Zhao et al. 2006). Biological responses of mast cells depend on the balance between positive and negative signals that are generated in FcR complexes (Malbec and Daeron 2007). Another level of communication with other cells and pathogen antigens can be achieved by mast cells via expression of TLRs (Dawicki and Marshall 2007). Additional level of complexity and further involvement of mast cells in immune system are demonstrated by fact that they can behave and function like professional APCs. First, they can express MHC class I and class II; second, they facilitate antigen presentation by expression of co-stimulatory molecules such as CD80 and CD86; and third, they can migrate to lymph nodes, where they further affect T cells gathering (Frandji et al. 1996; Hershko and Rivera 2010; Wang et al. 1998). Moreover, close interaction between mast cells and professional APCs such as DCs and LCs is essential for APCs to migrate, maturate, and present the antigen. This effect is linked to TNF-α and histamine produced by mast cell as well as their ability to accumulate exogenous antigens within endosomes (Kitawaki et al. 2006; Skokos et al. 2003; Suto et al. 2006). Recently, it has been shown that mast cells can shape and regulate immune response by ability to induce immune tolerance. A key player in this tolerance induction is anti-inflammatory cytokine IL-10 and to the laser extent transforming growth factor (TGF)-β released by mast cells. IL-10 has been shown to inhibit inflammatory skin reaction in contact hypersensitivity and UV-induced immunosuppression (Biggs et al. 2010; Byrne et al. 2008). The latter can be explained by UV effect on inhibition of germinal center formation and T helper (Th) function which in combination with IL-10 derived from mast cells creates immunosuppressive environment (Chacon-Salinas et al. 2011). Mast cells were shown to increase number of regulatory T cells (CD4^+^CD25^+^Foxp3^+^) via TGF-β-dependent mechanism (Zhang et al. 2010). The ability of mast cells to induce immunosuppression and angiogenesis as well as rearrangement of extracellular matrix is pivotal for their role in cancer. In skin cancer caused by UV irradiation, mast cells can be effectively attracted by upregulation of Kit receptor and tumor derived stem-cell factor (SCF) (Huttunen et al. 2002). The importance of Kit–SCF interaction for carcinoma perseverance was further documented in hepatocarcinoma, where increase in number of CD11b positive myeloid-derived suppressor cells and regulatory T cells as well as their IL-9 production was observed (Huang et al. 2008; Yang et al. 2010).

### T Cell Subsets

It is not a common knowledge that the skin is a reservoir of approximately 20 billion T cells, nearly twice the number present in the entire blood volume (Clark et al. 2006). Initially, the perception of skin immunosurveillance was based on T cells that migrate between skin-draining lymph nodes and peripheral tissues. Local defective migration of specific T cells rather than systemic decline in T-cell-mediated immunity is responsible for weaker DTH responses to bacterial, fungal, and viral antigens (Agius et al. 2009). Studies show that decrease in TNF-α secretion by macrophages inhibits activation of endothelial cells by suppression of E-selectin, vascular cell adhesion molecule, and ICAM expression, thus transmigration of T cells into the skin (Agius et al. 2009). However, the resident rather than recruited T cells creates skin immune homeostasis. T cell skin homing properties are obtained after imprinting process based on contact with skin DCs and mesenchymal cells (Edele et al. 2008; Mora et al. 2005). Some significant role in this process is attributed to vitamin D (Sigmundsdottir and Butcher 2008). Vitamin D has been found to inhibit effector T cell reactivity and induce regulatory T cells and their homing to sites of inflammation based on its critical role in AMPs production (Baeke et al. 2011). Epidermal CD8^+^ αβ T cells are of memory phenotype (Bos et al. 1987) and live among keratinocytes with preferential localization near LCs (Foster et al. 1990). Equal numbers of CD4^+^ and CD8^+^ T cells restricted to capillaries and the epidermal–dermal junction are characteristic for dermis (Mueller et al. 2013; Nomura et al. 2014). Most of them are memory cells expressing cutaneous lymphocyte-associated antigen. Skin memory T cells hold strategic position and create the first line of defense against pathogen challenge (Schenkel and Masopust 2014). They express CD103 and very late antigen 1 and after infectious recall undergo homeostatic proliferation (Gebhardt et al. 2009). Th17 together with Th1 and Th2 cells are important effector cells in inflammatory skin pathology such as allergic inflammation (Boyman et al. 2004; Conrad et al. 2007; Honda et al. 2013; Kim et al. 2013) or psoriasis (Conrad et al. 2007). IL-17 together with IL-22 produced by Th17 cells have well-documented role in psoriasis by inducing abnormal differentiation of keratinocytes. Nevertheless, Th17 cells protect skin from bacteria and fungi such as *Candida albinos, Klebsiella pneumonia,* and *Staphylococcus aureus* (Kashem et al. 2015; Kurebayashi et al. 2013). The magnitude of immune responses in skin is efficiently controlled by regulatory T cell (Tregs). They comprise of 5–10% of all resident skin T cells (Clark et al. 2006; Vukmanovic-Stejic et al. 2008). Together with other resident T cells, Tregs actively circulate between the skin and lymph nodes not only during immune response but also in the steady state (Clark 2010; Tomura et al. 2010). They can regulate T cell responses, function of APCs such as DCs and macrophages as well as neutrophil accumulation during early stages of inflammation (Richards et al. 2010; Schwarz and Schwarz 2010; Tiemessen et al. 2007). Tregs have been shown to induce anti-inflammatory functional profile in macrophages and inhibit macrophage TNF-α production (Tiemessen et al. 2007). It has been well documented that immunosuppression by Tregs is directly related to cancer. Primary and metastatic melanoma contain significant numbers of suppressor T cells, and in human squamous cell carcinoma of the skin, 50% of T cells are Foxp3 positive (Ahmadzadeh et al. 2008; Clark et al. 2008; Kaporis et al. 2007). Topical treatment with the TLR7 agonist increased E-selectin expression and reduced function and abundance of Tregs (Clark et al. 2008). Foxp3^+^ Treg depletion induced partial regression of established B16 melanoma in mice and accumulation of cytotoxic CD8^+^ T cells (Klages et al. 2010). Tregs were shown to inhibit Fas ligand-induced innate and adaptive tumor immunity in similar model and their removal improved tumor rejection (Simon et al. 2007).

Unconventional or innate resident T cells belong to γδ T cells and NKT cells (Hayday and Tigelaar 2003; Kronenberg 2005). Unlike naïve αβ T lymphocytes, skin γδ T cells reside in the epidermis in a pre-activated state. In mice, 90% of all T cells in epidermis are Vγ5^+^ dendritic epidermal T cells (Bergstresser et al. 1985). They provide immunoregulation and control over αβ T-cell-mediated inflammation (Boyden et al. 2008; Girardi 2006). In contrast to αβ T cells, γδ T cells inhibit skin tumor responses (Girardi et al. 2001, 2002; Roberts et al. 2007). Human γδ T cells are attributed to the regulation of epithelial homeostasis, cutaneous malignancy, and contact hypersensitivity (Holtmeier and Kabelitz 2005). Recent finding shows that IL-23 responsive dermal γδ T cells are the major IL-17 producers and may represent a novel target for the treatment of psoriasis (Cai et al. 2011). Moreover, skin γδ T cells act as primary responders to damage and wound repair due to their ability to produce growth factors participating in wound repairing (Toulon et al. 2009). Skin γδ T cells are able to produce certain AMPs such as cathelicidins allowing antimicrobial defense (Agerberth et al. 2000). The role in antimicrobial immune responses is also attributed to skin invariant NKT cells which can recognize bacterial glycolipids (Kronenberg 2005). Self-derived glycolipids recognized by CD1d-restricted NKT cells might activate keratinocytes and induce tissue pathology such as psoriatic plaques and allergic contact dermatitis (Bonish et al. 2000; Gober et al. 2008; Nickoloff et al. 2000). Activated NKT cells can maintain high levels of TNF-α and increase DCs migration from the skin to draining lymph nodes in a mouse model of hypersensitivity (Gorbachev and Fairchild 2006).

## Conclusion and Future Remarks

Current knowledge on immune-competent cells in the skin highlights the importance of the skin as a part of lymphatic system. The immune reactions that take place in periphery organ such as skin are equally important then those occurring within classical lymphoid organs in protection from the treath. In the era of vaccination and growing awareness of cancer, autoimmunity, and aging processes, the knowledge of skin immunity is of principal significance. Of high importance to our understanding of the skin immune response is the realization that this organ is characterized by diverse and low oxygen pressure (Grillon et al. 2012). Thus, more attention should be paid to environmental factors such as hypoxia, the low oxygen pressure that is divergent in different layers of the skin and governs immune reactions in this organ. Immune cells respond to low oxygen pressure in a different ways depending on the cell type. Skin hypoxia promotes the survival, recruitment, and activation of innate immune cells, whereas inhibits effector lymphocyte functions. Transcription factor hypoxia-inducible factor (HIF)-1α is the key regulator of cellular adaptation to hypoxia. HIF-1α plays a role in bactericidal capacity of macrophages and neutrophils. It has been shown that HIF-1α regulates the production of cathelicidin by keratinocytes, thus is crucial for their antibacterial function (Peyssonnaux et al. 2008). In case of DCs, hypoxic microenvironment exerts a sharp pressure to allow pro-inflammatory and antimicrobial functions. Hypoxia has also a strong influence on sugar-binding properties by lectins which are imperious in immune recognition mechanisms, and in case of galectin-1 enhances carbohydrate binding. Expression of Langerin, a C-type lectin, creates a main characteristic for different subpopulation of skin DCs including LCs and a common antigen/pathogen recognition receptor (Stoitzner and Romani 2011). Another imperious factor that should be considered in our understanding of skin immunity is aging. Skin aging is a multifactorial process that involves defect in the function of skin immune cells. Increase in cutaneous infections and cancer becomes prominent in older humans. It has been suggested that both acquired and innate immunity are compromised with age. A substantial role in suppression of innate and acquired immune responses is attributed to Tregs. Older individuals have increased number of Tregs in normal skin. In general, older subjects are characterized by reduced cutaneous DTH responses and decreased infiltration of T cells (Castle et al. 1990; Toichi et al. 1997; Vissinga et al. 1990).

Understanding the mechanisms of skin immunity in different environmental settings will allow better therapeutic approaches both in dermatology and cosmetical industry. Recently, there is a growing interest in new and especially natural compounds with antioxidant and immunity boosting or diminishing properties that are promising in prevention from skin disease and premature aging.
